# Blockade of STAT3 Causes Severe In Vitro and In Vivo Maturation Defects in Intestinal Organoids Derived from Human Embryonic Stem Cells

**DOI:** 10.3390/jcm8070976

**Published:** 2019-07-04

**Authors:** Kwang Bo Jung, Ohman Kwon, Mi-Ok Lee, Hana Lee, Ye Seul Son, Omer Habib, Jung-Hwa Oh, Hyun-Soo Cho, Cho-Rok Jung, Janghwan Kim, Mi-Young Son

**Affiliations:** 1 Stem Cell Convergence Research Center, Korea Research Institute of Bioscience and Biotechnology (KRIBB), Daejeon 34141, Korea; 2 Department of Functional Genomics, KRIBB School of Bioscience, Korea University of Science and Technology, Daejeon 34113, Korea; 3 Department of Pathology, College of Medicine, Kyung Hee University, Seoul 02447, Korea; 4 Department of predictive toxicology, Korea Institute of Toxicology, Daejeon 34114, Korea

**Keywords:** human intestinal organoid, STAT3, in vitro maturation, CRISPR/Cas9, human embryonic stem cell

## Abstract

Human intestinal organoids (hIOs), which resemble the human intestine structurally and physiologically, have emerged as a new modality for the study of the molecular and cellular biology of the intestine in vitro. We recently developed an in vitro maturation technique for generating functional hIOs from human pluripotent stem cells (hPSCs). Here, we investigated the function of STAT3 for inducing in vitro maturation of hIOs. This was accompanied by the tyrosine phosphorylation of STAT3, whereas treatment with pharmacological inhibitors of STAT3 suppressed the phosphorylation of STAT3 and the expression of intestinal maturation markers. We generated and characterized STAT3 knockout (KO) human embryonic stem cell (hESC) lines using CRISPR/Cas9-mediated gene editing. We found that STAT3 KO does not affect the differentiation of hESCs into hIOs but rather affects the in vitro maturation of hIOs. STAT3 KO hIOs displayed immature morphologies with decreased size and reduced budding in hIOs even after in vitro maturation. STAT3 KO hIOs showed markedly different profiles from hIOs matured in vitro and human small intestine. Additionally, STAT3 KO hIOs failed to maintain upon in vivo transplantation. This study reveals a core signaling pathway consisting of STAT3 controlling the in vitro maturation of hIOs derived from hPSCs.

## 1. Introduction

Human intestinal organoids (hIOs) are stem cell-derived, three-dimensional (3D) multicellular structures that closely mimic the physiology, functions, and cell organizations of intestinal tissues in vivo [1,2]. hIOs have been previously derived from human pluripotent stem cells (hPSCs) or native biopsies, both of which are known to share similar properties, including the specialized intestinal epithelial cell types within hIOs and crypt-villus structures similar to those of the intestinal epithelium [3,4]. hIOs are a promising tunable source for intestinal developmental and disease modeling, drug absorption, and toxicity testing, and gut microbiota interaction models. hPSC-derived hIOs create additional layers of diverse mesenchymal cells that are highly representative of the intestines in vivo [5]. However, hPSC-derived hIOs have several limitations, such as immature, fetal-like transcriptional profiles and limited functionalities [6,7]. Until recently, the cellular and functional maturation of hPSC-derived hIOs required in vivo transplantation into immunocompromised mice [8] or in vitro maturation by co-culture with immune cells or by exposure to IL-2 as in our previous study [9].

We previously showed that the simulation of the human intestinal environment by co-culturing hIOs derived from human embryonic stem cells (hESCs) and human induced pluripotent stem cells (hiPSCs) with human T lymphocytes induced the in vitro maturation of hIOs, where interleukin-2 (IL-2) was identified as the major promoting factor of maturation. As a result, the hIOs matured in vitro contain all functional specialized intestinal epithelial cell types, including enterocytes, goblet cells, Paneth cells, and enteroendocrine cells. Next generation sequencing (NGS)-based transcriptome analysis (RNA sequencing) also confirmed that the whole gene expression levels were similar to those in the adult human small intestine (hSI). Interestingly, the effect of co-culture and IL-2 on the in vitro maturation of hIOs is mediated by the activation of STAT3, a transcription factor activated by cytokine-induced intracellular signals. STAT3 is known to play an important role in intestinal homeostasis [10,11], IL-22-mediated intestinal regeneration [12], and intestinal stem cell survival [13]. The activation of innate responses mediated by STAT3 signaling contributes to the amelioration of colitis [14]. However, STAT3 mediates the activation of acquired immune responses, which seem to play a role in the pathogenesis of colitis by promoting the survival of pathogenic T cells [15]. Therefore, the pharmacological regulators of STAT3 maintaining STAT3 homeostasis and adequate STAT3 levels may be promising therapeutic candidates for intestinal disorders.

However, thus far the exact requirements of STAT3 for the in vitro maturation of hPSC-derived hIOs have not been fully elucidated. Therefore, in this study, we tested various specific pharmacological inhibitors that inhibit STAT3 signaling and established STAT3 knockout (KO) hESC lines using a clustered regularly interspersed short palindromic repeat (CRISPR)-CRISPR-associated protein 9 (Cas9) genome editing system [16]. We demonstrated that treatment with STAT3 inhibitors reduced the expression of intestinal maturation markers in hIO epithelium, which correlated with reduced activation of STAT3 phosphorylation. Hence, we found that hIOs derived from STAT3 KO hESCs displayed a severe disadvantage in in vitro maturation and a defect in maintenance in vivo after transplantation. STAT3 inhibitor and KO studies helped elucidate the signaling network involved in hIO maturation and provided much-needed information for advancing the generation of hPSC-derived hIOs, which are more similar to native intestinal tissues in vivo than immature hIOs, which did not undergo in vitro maturation.

## 2. Materials and Methods

### 2.1. Chemicals And Reagents

Cell culture medium (RPMI 1640, advanced DMEM/F12 medium), defined fetal bovine serum (dFBS), and B27 were acquired from Thermo Fisher (Waltham, MA, USA). Activin A, fibroblast growth factor 4 (FGF4), WNT3A, epidermal growth factor (EGF), Noggin, and recombinant human IL-2 were purchased from R&D Systems (Minneapolis, MN, USA). R-Spondin 1 was purchased from PeproTech Inc (Rocky Hill, NJ, USA). Specific inhibitors of STAT3, including S3I-201 or Stattic, phorbol myristate acetate (PMA), and calcium ionophore A23187 were purchased from Sigma-Aldrich (St. Louis, MO, USA).

### 2.2. Differentiation And Culture of hIOs From hESCs

The H9 hESC line was purchased from the WiCell Research Institute (Madison, WI, USA). Human T lymphocytes (Jurkat T cells) were obtained from the American Type Culture Collection (ATCC; Manassas, VA, USA). Fibroblasts and hESCs were cultured, as described previously [17]. hIOs were generated from hESCs, as described previously [18]. Briefly, 80 to 90% confluent hESCs were differentiated into definitive endoderm (DE) through treatment with 100 ng/mL Activin A for 3 days in RPMI 1640 medium with increasing concentrations of 0%, 0.2%, and 2% dFBS. For differentiation into hindgut spheroids, DE cells were cultured in hindgut differentiation medium containing RPMI 1640, 500 ng/mL FGF4, and 500 ng/mL WNT3A for 4 days. Free-floating hindgut spheroids were transferred into three-dimensional cultures in Matrigel (BD Biosciences, San Diego, CA, USA) and cultured in hIO medium containing 1X B27, 100 ng/mL EGF, 500 ng/mL R-Spondin 1 in the presence of 100 ng/mL Noggin, which was replaced every two days. hIOs were passaged every 10 to 14 days thereafter, following the protocol described previously [19]. In vitro maturation experiments were conducted by IL-2 treatment or co-culture with human T lymphocytes as described previously [9]. A Transwell insert (pore size 0.4 μm, Corning, NY, USA) on which hIOs had been embedded within Matrigel (BD Biosciences) was placed into the well of a 12-well plate containing stimulated Jurkat T cells (5 × 10^4^/cm^2^) with 50 ng/mL PMA and 500 ng/mL A23187 for 3 h and cultured for 2 passages. The well-differentiated and characterized hIOs were treated with freshly prepared 1–8 ng/mL IL-2 daily to hIO medium for 2 passages. At least three biological replicates for each differentiation experiment were used. For STAT3 signaling, hIOs were treated with S3I-201 (10 μM) or Stattic (1 μM) and added to hIO culture medium for 10 days. The surface area using horizontal cross-sections of hIOs was calculated to determine the size of hIOs.

### 2.3. Generation And Validation of STAT3 KO hESC Lines

RNA was transcribed in vitro using the MEGAshortscript T7 kit (Ambion, Invitrogen) according to the manufacturer’s manual. Templates for a synthetic guide RNA (sgRNA) were generated by annealing and extension of two complementary oligonucleotides (Supplementary Table S1). Transcribed RNA was purified by a MEGAclear Transcription Clean-Up Kit (Ambion). Purified RNA was quantified by spectrometry. H9 hESCs expressing Cas9 under the control of a tetracycline-responsive element were dissociated into single cells using gentle cell dissociation reagent (STEMCELL Technologies, Cambridge, MA, USA). Cells (1 × 10^6^) resuspended in Nucleofector solution were electroporated with 40 μg of in vitro transcribed sgRNA by using an Amaxa P3 Primary Cell 4D-Nucleofector Kit (Lonza, Walkersville, MD, USA). Cells were maintained in the presence of doxycycline. After 3 days, cells were replated as single cells at a very low density on Laminin 521-coated plates in Essential 8 medium (Thermo Fisher Scientific, Voltam, MA, USA) supplemented with Rho kinase (ROCK) inhibitor (Y-27632, Stemgent, MA, USA). Individual colonies were picked and expanded. Genomic DNA was then extracted using QuickExtract (Epicenter, Madison, WI, USA) according to the manufacturer’s instructions. The target region was amplified using Phusion polymerase (New England Biolabs Inc., Ipswich, MA, USA) (Supplementary Table S2) and used for library construction. PCR amplicons were subjected to paired-end read sequencing using Illumina MiSeq (Illumina, San Diego, CA, USA). For T7 endonuclease I (T7E1) assay, 200 ng of DNA heteroduplexes of PCR products obtained from WT and STAT3 KO cells were incubated with 10 U of T7E1 at 37 °C for 15 min in a reaction volume of 20 μL. The reactions were analyzed by 2% agarose gel electrophoresis.

### 2.4. Quantitative Real-Time PCR (qPCR)

Total RNA was extracted from hESCs and hIOs using an RNeasy Kit (Qiagen, Valencia, CA, USA) and reverse-transcription was done using a Superscript IV First-Strand Synthesis System Kit (Invitrogen) as described previously [20]. Quantitative real-time PCR (qPCR) qPCR was performed three times independently using SYBR green PCR Master Mix (Applied Biosystems, Foster City, CA, USA) on a 7500 Fast Real-time PCR system (Applied Biosystems) as described previously [21]. The primers used in this study are listed in Supplementary Table S3.

### 2.5. Immunofluorescence And Hematoxylin-Eosin (H&E) Staining

Immunofluorescence staining was performed as described previously [22]. hIOs were fixed in 4% paraformaldehyde (PFA) and cryoprotected in sucrose. hIOs were frozen in optimal cutting temperature (OCT) compound (Sakura Finetek, Tokyo, Japan), cut at 20 μm using a cryostat microtome at −20 °C and permeabilized with 0.1% Triton X-100. After being blocked with 4% bovine serum albumin (BSA), samples were incubated with primary antibodies (Supplementary Table S4) at 4 °C overnight, followed by incubation with the corresponding Alexa Fluor 488-, Alexa Fluor 594-, or Alexa Fluor 647-conjugated secondary antibodies for 1 h at room temperature. Paraffin sections of hSI (Jejunum tissue slide, Novus Biologicals NBP2-30201, Centennial, Colorado, USA) were deparaffinized, followed by antigen retrieval in citrate buffer, and stained in a similar fashion to the OCT sections. The nuclei were stained with diamidino-phenylindole (DAPI, 1 mg/mL, Invitrogen). Samples were visualized with an Axiovert 200M microscope (Carl Zeiss, Gottingen, Germany) and a fluorescence microscope (IX51, Olympus, Japan). Histological sections from kidneys were stained with H&E using a standard staining protocol.

### 2.6. Transmission Electron Microscopy (TEM)

hIOs were harvested and fixed overnight with 2.5% glutaraldehyde in 0.1 M phosphate buffer (pH 7.3) at room temperature. After fixation, hIOs were postfixed with 1% OsO_4_ for 1 h at 4 °C while protected from light. Dehydration was performed in an ethanol and propylene oxide series before embedding with EPON812 (Electron Microscopy Sciences, Hatfield, PA, USA). hIOs were polymerized using pure resin at 70 °C for 2 days. Ultrathin sections (70 nm) were obtained using an ultramicrotome (UltraCut-UCT, Leica, Wetzlar, Germany) and were collected on 150 mesh copper grids. hIOs were stained with 2% uranyl acetate for 15 min and lead citrate for 5 min. The BIO-TEM images were recorded with transmission electron microscopy (TEM) (Tecnai G2 Spiri TWIN microscope, Thermo Fisher Scientific) at 120 kV.

### 2.7. Transcriptome Analysis By Microarray

The microarray experiments were performed according to the manufacturer’s instructions using Whole Human Genome Microarray 4 × 44 K (Agilent Technology, Santa Clara, CA, USA), as previously described [23]. The gene expression data were processed using GeneSpring software (Agilent) and normalized using global scale normalization. A hierarchically clustered heat map was generated using MeV v4.9.0 software. Differentially expressed gene (DEG) selection and gene clustering were performed through a web-based application (https://amp.pharm.mssm.edu/biojupies/) [24]. Gene ontology analysis related to the biological process involving genes in each cluster was performed through the Reactome FI app of the open source software platform Cytoscape (Version 3.6.1, https://cytoscape.org/). The key pathways controlling the expression of each cluster gene were found by analyzing the highly interconnected regions of the Reactome Functional Interactome (FI) by the MCODE application of Cytoscape (Version 3.6.1), an open source software platform. The expression level of the intestinal marker, defense response, and transporter ME genes are listed in Supplementary Table S5.

### 2.8. Transplantation

NOD-SCID IL-2Rγnull (NSG) mice (8–12 weeks old) (Jackson Laboratories, Bar Harbor, ME, USA) were placed within a standard animal housing facility at a constant temperature (20–22 °C) under a 12-h light:12-h dark cycle. All animal experiments were carried out after approval by the Institutional Animal Care and Use Committee (IACUC) of KRIBB (approval No: KRIBB-AEC-18210). For in vivo transplantation, hIOs were cultured with hIO medium consisting of 100 ng/mL EGF [8]. hIOs were incubated by embedding into purified collagen type I (rat tail collagen; BD Biosciences) at 37 °C for 12 h before transplantation. Transplantation of hIOs into the kidney capsule was previously described [9]. Mice were anesthetized with 2% isoflurane (Butler Schein, Dublin, OH, USA), and the left side of the mouse was then prepared using povidone-iodine and isopropyl alcohol in a standard fashion. A left subcostal incision was used to expose the kidney. The hIOs in the collagen plug were then transplanted into the capsular region of the kidney. The kidney was then returned to the peritoneal space. The skin was closed with a running suture, and the mice were warmed with a heating pad until they had recovered fully from the anesthesia. Mice were monitored regularly and euthanized humanely 2 to 4 weeks after transplantation, and the xenografts were isolated for analysis.

### 2.9. Western Blotting

The hIOs were harvested and lysed with RIPA buffer (Sigma-Aldrich) supplemented 1× PMSF (Sigma-Aldrich), 1 mM protease inhibitor, and 1× phosSTOP (Roche, Indianapolis, IN, USA). Then, 20 μg of total protein was separated by electrophoresis on pre-cast gels (4–20% gradient; Bio-Rad Laboratories, Hercules, CA, USA) and transferred. After blocking, the membranes were incubated with the appropriate primary antibodies (anti-phospho STAT3 (Y705), 1:2000 (Abcam, Cambridge, UK); anti-STAT3, 1:1000 (Abcam); anti-β-actin, 1:5000 (Sigma-Aldrich)) at 4 °C overnight. After washing, the membranes were incubated with secondary antibody conjugated with HRP (goat anti-mouse IgG-HRP, 1:2000 (Santa Cruz Biotechnology, CA, USA); goat anti-rabbit IgG-HRP, 1:2000 (Santa Cruz Biotechnology)). The band was detected using the luminescent image analyzer LAS-3000 (Fuji Photo Film GMBH, Tokyo, Japan)

### 2.10. Fluo-4 AM Assay

The hIOs were treated for 1 h with 5 μM Fluo-4 acetoxymethylester (Fluo-4 AM; Molecular Probes, Eugene, Oregon, USA) and washed with Ca^2+^-free isotonic buffer (140 mM NaCl, 5 mM KCl, 10 mM HEPES, 2 mM MgCl_2_, 5.5 mM D-Glucose). The hIOs were mounted on a confocal microscope (FV1000 Live; Olympus) and stimulated with 50 mM glucose (Sigma-Aldrich) in Ca^2+^-free isotonic buffer. hIOs were excited at 488 nm, and the signal emitted at 505 to 530 nm was recorded. The fluorescence intensity of the region of interest (ROI) was calculated using FV1000 software.

## 3. Results

### 3.1. Treatment With STAT3 Inhibitors Abrogated The In Vitro Maturation Of hESC-Derived hIOs

**Figure 1:**
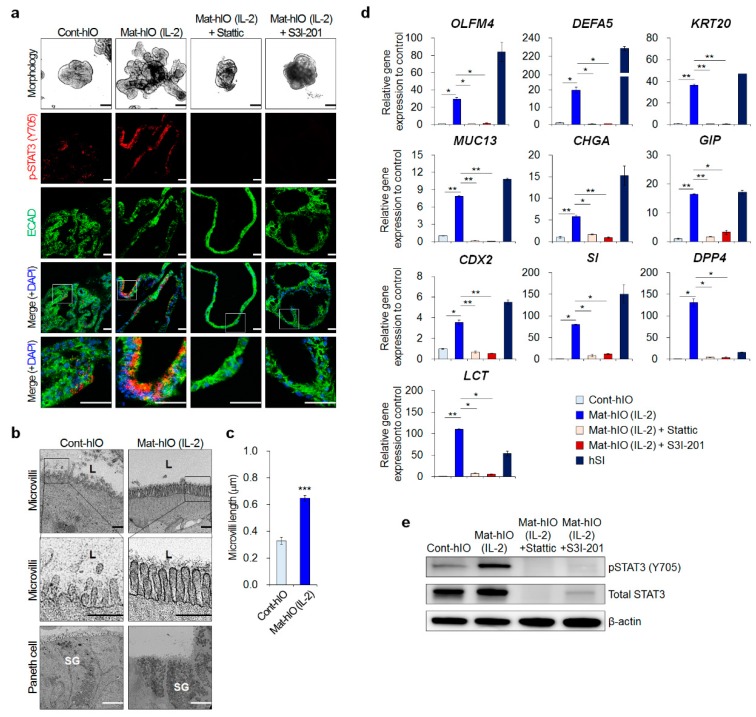
In vitro maturation of hPSC-derived hIOs is accompanied by the activation of STAT3 signaling. (**a**) Representative morphologies and immunofluorescence staining with p-STAT3 (Y705) and ECAD in Cont-hIOs, and IL-2 treated Mat-hIOs in the presence or absence of STAT3 inhibitors, including Stattic (1 μM) or S3I-201 (10 μM). Black scale bar, 200 μm. White scale bar, 50 μm. (**b**) Transmission electron microscopy images for epithelial characterization of control hIOs and IL-2-treated Mat-hIOs. ‘L’ indicates lumen, and ‘SG’ indicates secretory granules. Black scale bar, 500 nm. White scale bar, 5 μm. (**c**) The length of microvilli (after three passages of maturation, n = 20 per group). (**d**) qPCR analysis of intestinal cell type-specific maturation markers in Cont-hIOs, IL-2 treated Mat-hIOs in the presence or absence of STAT3 inhibitors (Stattic, S3I-201), and hSI (hIOs passage 4, n = 3 per group). Data are presented as the mean value of replicates ± standard error of the mean (SEM). *** *p* < 0.001, ** *p* < 0.01, and * *p* < 0.05 according to Student’s *t*-test. (**e**) Western blot analysis of p-STAT3 (Y705) and STAT3 in Cont-hIOs and IL-2 treated Mat-hIOs in the presence or absence of STAT3 inhibitors.

Our previous studies showed that treatment with STAT3 inhibitors alters the morphology of hIOs with reduced budding and surface area of hIOs [9]. In this study, we aimed to elucidate how STAT3 regulates the in vitro maturation process of hIOs in greater detail. As described in our previous report [9], IL-2 treatment over two passages results in the formation of mature hIOs (Mat-hIOs) (Figure 1a). TEM analysis demonstrated that the brush border in Mat-hIO epithelium was well developed and Mat-hIO epithelium had longer microvilli than those of control hIO epithelium (Cont-hIOs) (Figure 1b). In accordance with the microvilli length in the human adult small intestine, which ranges from …0.6 to …1.7 μm [25,26], the microvilli of Mat-hIO epithelium were approximately 0.647 ± 0.021 μm in length (Figure 1c). The microvilli length was correlated with a significantly higher expression of brush border enzymes, such as dipeptidyl peptidase IV (*DPP4*), sucrase isomaltase (*SI*), and lactase (*LCT*), in Mat-hIOs compared to Cont-hIOs (Figure 1d). Furthermore, functional Paneth cells with secretory granules, which are essential for intestinal stem cell maintenance [27] and are a representative cell type for mature hIOs [6,8,9], were clearly recognized and more frequent in Mat-hIOs compared to Cont-hIOs by TEM analysis (Figure 1b). qPCR analysis also showed that Mat-hIOs contained mature and functional Paneth cells, as evidenced by the increased expression of antimicrobial peptides (*DEFA5*) secreted by mature Paneth cells, similar to hSI (Figure 1d).

We previously demonstrated the involvement of the STAT3 signaling pathway in hIO maturation [9]. Intense phospho-STAT3 immunostaining for detecting phosphorylation at the tyrosine 705 (Y705) residue was visualized in Mat-hIOs compared to Cont-hIOs (Figure 1a), indicating STAT3 signaling activation in the epithelial maturation of hIOs. This phosphorylation was completely blocked by the addition of the specific STAT3 inhibitors Stattic and S3I-201 (Figure 1a). Consistently, the phosphorylation levels of STAT3 were significantly increased in Mat-hIOs, but almost diminished after treatment with the STAT3 inhibitors (Figure 1e). Most importantly, treatment with the STAT3 inhibitors completely ablated the expression of intestinal cell type-specific maturation markers, including mature intestinal stem cell marker (*OLFM4*), mature Paneth cell markers (*DEFA5*), mature goblet cell marker (*MUC13*), mature enteroendocrine cell markers (*CHGA* as a general enteroendocrine marker *GIP* as a marker of enteroendocrine K cells), mature enterocyte markers (*SI*, *DPP4*, *LCT*), and other intestinal markers (*CDX2*, *KRT20*) (Figure 1d). These results indicated that the activation of STAT3 is critical for maintaining the mature state of multiple intestinal epithelial cell types and that the specific STAT3 inhibitors Stattic and S3I-201 exert an anti-maturation effect on the epithelium of hIOs.

### 3.2. Generation And Validation of STAT3 KO hESC Lines

**Figure 2:**
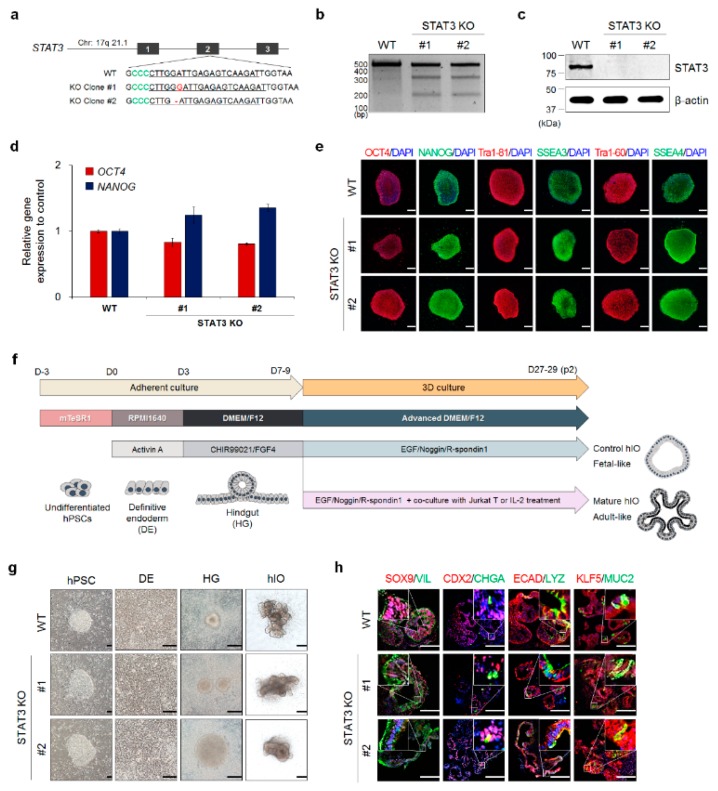
Generation and characterization of the STAT3 KO hESC line using CRISPR-Cas9 genome editing. (**a**) Schematic representation of *STAT3* gene structure and the mutant genotype of STAT3 KO hESC lines. Exons of *STAT3* are shown by black rectangles, and targeting sequences are indicated at the bottom of the *STAT3* gene structure. The nucleotide sequences of sgRNA are underlined, the PAM (NGG) sequences are represented in green letters, and the insertion and deletion mutations in the *STAT3* gene are represented by red letters and dashes. (**b**) T7 endonuclease 1 (T7E1) assay for mutation verification of STAT3 KO hESC lines. Genomic PCR products from each clone and heteroduplex of the control and individual clones were digested by T7E1. (**c**) Western blot analysis of STAT3 in WT and STAT3 KO hESC lines. (**d**) qPCR analysis of pluripotency markers in undifferentiated H9, WT, and STAT3 KO hESC lines. Data are presented as the mean value of replicates ± SEM. (after two passages of maturation, n = 3 per group). (**e**) Immunofluorescence analysis for the pluripotency markers OCT4, NANOG, TRA-1-60, TRA-1-81, SSEA-3, and SSEA-4. Scale bar, 200 μm. (**f**) Schematic representation of hPSC differentiation into hIOs and in vitro maturation of hIOs. (**g**) Representative morphologies during the differentiation process from hESCs to definitive endoderm (DE), hindgut (HG), and hIOs. Scale bar, 200 μm. (**h**) Immunofluorescence analysis for intestine-specific markers (SOX9, CDX2, and KLF5); the enterocyte marker, villin 1 (VIL); the enteroendocrine cell marker, chromogranin A (CHGA), the goblet cell marker, mucin 2 (MUC2); the Paneth cell marker, lysozyme (LYZ); and the epithelial marker, ECAD. Scale bars, 200 μm.

To investigate the role of STAT3 in the in vitro maturation of hPSC-derived hIOs, we attempted to generate STAT3 KO hESC lines. For the efficient generation of insertion/deletion (in/del) mutations mediated by CRISPR/Cas9, we designed a single sgRNA targeting the region immediately downstream of the start codon in the second exon of the *STAT3* gene (Figure 2a). The in vitro transcribed sgRNA was transfected into an hESC line expressing Cas9 under the control of a tetracycline-responsive element (TRE) via electroporation in the presence of doxycycline (Dox). At 72 h post-transfection, cells were dissociated and replated as single cells at a very low density in hESC medium supplemented with Rho kinase (ROCK) inhibitor to obtain single cell-derived clones. After picking and expanding individual clones, we confirmed the disruption of the *STAT3* targeted locus by deep sequencing (Figure 2a). As a result, two frameshifted clones were isolated, and a single nucleotide insertion (+G in *STAT3* KO clone #1) and deletion (-G in *STAT3* KO clone #2) were confirmed. In addition, the T7E1 assay clearly showed enzymatically digested products, which mean the successful introduction of in/del mutations in the targeted region (Figure 2b). We also observed the complete absence of STAT3 protein expression in two selected STAT3 KO hESC lines (KO#1 and KO#2) by Western blotting analysis (Figure 2c). Thus, we concluded that STAT3 KO hESC lines were successfully generated by CRISPR-Cas9 genome editing.

We subsequently examined whether STAT3 KO hESC lines could maintain pluripotent characteristics by examining the expression of pluripotency markers. Similar to WT hESCs, two STAT3 KO hESC lines expressed *OCT4* and *NANOG* and immunostained positive for OCT4, NANOG, TRA-1-81, SSEA3, TRA-1-60, and SSEA4 (Figure 2d,e), demonstrating that STAT3 KO did not affect the pluripotency of hESCs. STAT3 KO hESC lines were induced to differentiate into hIOs using a conventional stepwise differentiation protocol to derive fetal-like Cont-hIOs from hPSCs (Figure 2f) [4,8]. WT and STAT3 KO hESC lines were efficiently differentiated into DE, hindgut, and hIOs with adequate lineage-characteristic morphologies (Figure 2g). We assessed the expression of intestinal transcription factors, including SOX9, CDX2, and KLF5, and intestinal cell type-specific markers, including villin 1 for enterocytes (VIL), chromogranin A for enteroendocrine cells (CHGA), lysozyme for Paneth cells (LYZ), and mucin 2 for goblet cells (MUC2), showing that Cont-hIOs, regardless of whether they were derived from WT or STAT3 KO hESC lines, contained all intestinal epithelial cell types (Figure 2h).

### 3.3. STAT3 KO hESC Lines Showed A Severe hIO Maturation Disadvantage In Vitro

**Figure 3:**
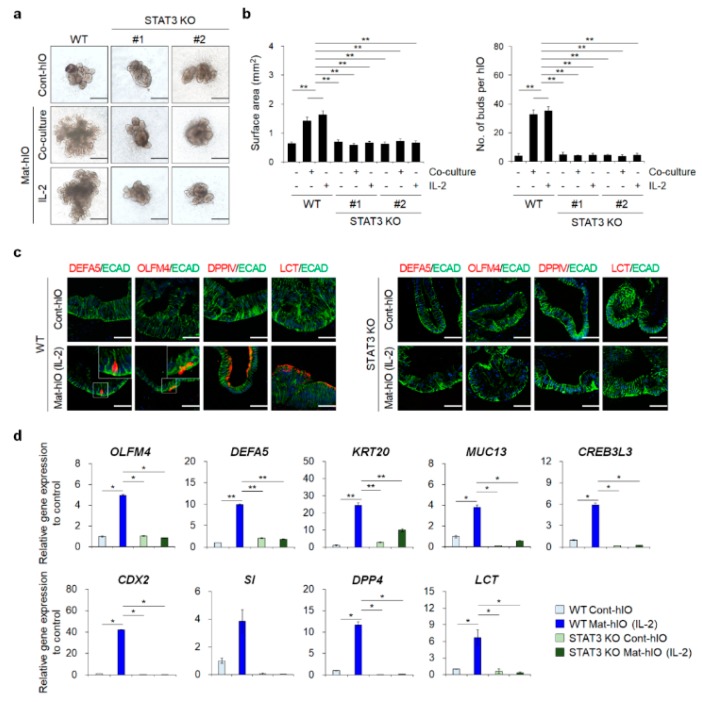
In vitro maturation of STAT3 KO hIOs. (**a**) Representative images of morphological changes during in vitro maturation of hIOs by co-culture with PMA/ionophore-stimulated Jurkat T or treatment with IL-2. Scale bar, 500 μm. (**b**) Quantitative assessment of the surface area of hIOs (left panel, after two passages of maturation, n = 5 per group) and the number of budding structures per single hIO (right panel, after two passages of maturation, n = 5 per group). Data are presented as the mean value of replicates ± SEM. *** *p* < 0.001, ** *p* < 0.01, and * *p* < 0.05 according to Kruskal–Wallis test. (**c**) Immunofluorescence staining of WT hIOs and STAT3 KO hIOs with an epithelial marker (ECAD) and mature intestinal markers (DEFA5, OLFM4, DPPIV, and LCT) following in vitro maturation. Scale bar, 50 μm. (**d**) qPCR analysis of intestinal maturation markers in WT Mat-hIOs and STAT3 KO Mat-hIOs by treatment with IL-2 (after three passages of maturation, n = 4 per group). Data are presented as the mean value of replicates ± SEM. *** *p* < 0.001, ** *p* < 0.01, and * *p* < 0.05 according to Student’s *t*-test.

To investigate the role of STAT3 in the in vitro maturation of hPSC-derived hIOs, we examined whether hIOs derived from these edited hESC lines could acquire intestinal maturation characteristics by assessing their morphologies and expression of intestinal maturation markers. Two passages after the induction of in vitro maturation by IL-2 treatment or co-culture with PMA/ionophore-stimulated Jurkat T lymphocytes, phenotypes of maturation were observed in WT hESC-derived hIOs (WT hIOs), which demonstrated an increased hIO size and an average number of buds per hIO (Figure 3a,b). The increase of the surface area of the hIOs indicated that the cell proliferation of the hIOs was enhanced, and the increase of the budding number showed that the number of intestinal stem cells and the differentiation capacity into intestinal epithelial cell types were enhanced. Concomitantly, the WT Mat-hIO epithelium exhibited positive staining for well-known intestinal maturation-related proteins, such as DEFA5 and OLFM4, and functional brush-border enzymes, such as DPP4 and LCT (Figure 3c). However, marked phenotypic differences were observed in the epithelium of STAT3 KO hESC-derived hIOs (STAT3 KO hIOs) even after in vitro maturation, which was also reflected in the reduced hIO size and the average number of buds per hIO (Figure 3a,b). We also examined the expression of DEFA5, OLFM4, DPP4, and LCT, which did not appear in the epithelium of STAT3 KO Mat-hIOs (Figure 3c), even though the markers of all the intestinal epithelial cell types were found in the epithelium of STAT3 KO Mat-hIOs (Supplementary Figure S1). These results were further confirmed by qPCR analysis of intestinal maturation markers (Figure 3d). After in vitro maturation by IL-2 treatment, STAT3 KO Mat-hIOs showed significantly lower expression levels of *OLFM4*, *KRT20*, *MUC13*, *CREB3L3*, *CDX2*, *SI*, *DPP4*, and *LCT*, similar to Cont-hIOs, compared to WT Mat-hIOs. To discriminate impaired in vitro maturation of hIOs from cell death, we performed co-staining with apoptotic cell death marker, cleaved caspase-3, and intestinal epithelial cell markers, OLFM4 or LYZ, respectively. The expression levels of OLFM4 and LYZ were significantly decreased in STAT3 KO hIOs and STAT3 inhibitor-treated hIOs compared to WT Mat-hIOs. However, the expression level of cleaved caspase-3 was not altered in STAT3 KO hIOs or STAT3 inhibitor-treated hIOs (Supplementary Figure S2). These results suggest that STAT3 KO severely impairs the in vitro maturation of the hIO epithelium, regardless of cell survival.

### 3.4. Transcriptome Comparisons Revealed That STAT3 Is Necessary for In Vitro hIO Maturation

**Figure 4:**
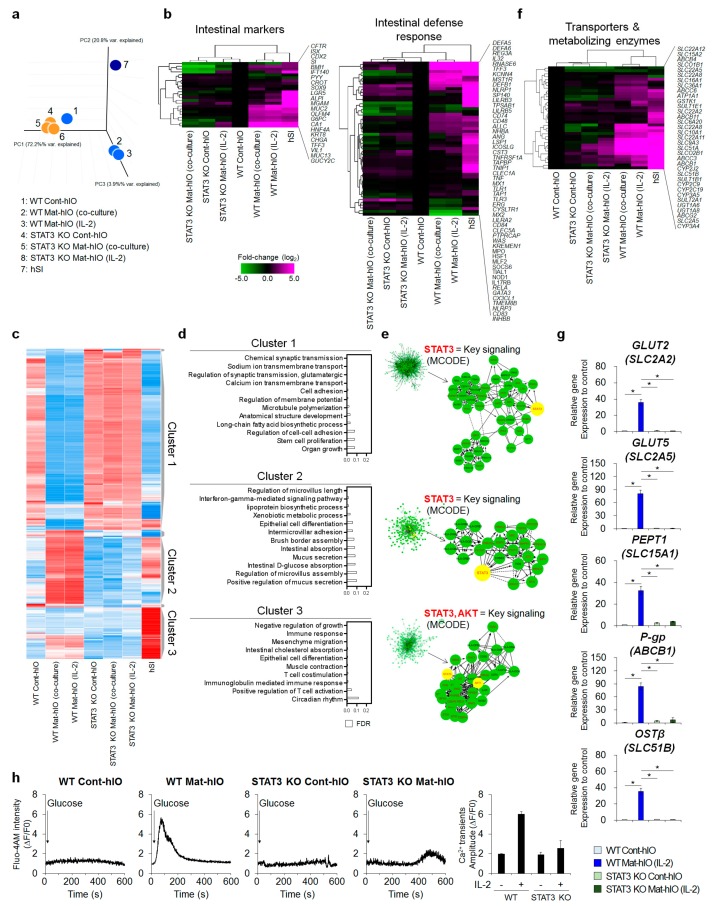
Transcriptomic analysis of STAT3 KO hIOs based on the microarray data. (**a**) Principal component analysis (PCA) of microarray datasets for WT hIOs and STAT3 KO hIOs with or without in vitro maturation by either IL-2 treatment or co-culture with stimulated Jurkat T lymphocytes and adult human small intestine (hSI). (**b**) Heatmaps and hierarchical clustering of genes involved in the intestinal markers and intestinal defense response in WT hIOs and STAT3 KO hIOs with or without in vitro maturation. Adult hSI was used as the control. Gene expression was normalized to that of WT Cont-hIOs (shown in black). Magenta indicates upregulated genes, whereas green indicates downregulated genes compared to those of the WT Cont-hIOs. (**c**) Heatmap of differentially expressed genes (DEGs) in WT hIOs and STAT3 KO hIOs with or without in vitro maturation conditions. Three clusters classified according to the expression pattern are displayed on the right side of the heatmap. (**d**) The representative Gene Ontology (GO) pathway of genes belonging to each cluster is indicated by a bar graph according to the false discovery rate (FDR) and *p*-value. (**e**) The key signaling pathway enriched from reactome FI networks by MCODE, which regulates the expression of each cluster gene. (**f**) Heatmaps and hierarchical clustering of genes involved in intestinal transporter and metabolizing enzymes. (**g**) qPCR analysis of transporter and metabolizing enzymes in WT hIOs and STAT3 KO hIOs with or without in vitro maturation by IL-2 treatment (after three passages of maturation, n = 3 per group). Data are presented as the mean value of replicates ± SEM. ** *p* < 0.01, and * *p* < 0.05 according to Student’s *t*-test. (**h**) Cytosolic Ca2+ transients induced by glucose treatment in real-time in WT hIOs and STAT3 KO hIOs with or without in vitro maturation by IL-2 treatment (after two passages of maturation, n = 3 per group).

To identify the genes that were differentially expressed upon the inhibition of STAT3 during the in vitro maturation of hIOs, we performed microarray analyses of WT Cont-hIOs, STAT3 KO Cont-hIOs, WT Mat-hIOs, and STAT3 KO Mat-hIOs, whose maturation was induced by either IL-2 treatment or co-culture with stimulated Jurkat T lymphocytes, as well as adult hSI. The seven samples were distinctly separated into three groups by principal component analysis (PCA) of the whole transcriptome (Figure 4a). WT Cont-hIOs, STAT3 KO Cont-hIOs, and STAT3 KO Mat-hIOs were closely clustered, whereas WT Mat-hIOs was separated from that group even though WT Mat-hIOs was not fully clustered with hSI. However, the expression profile of WT Mat-hIOs was most closely related to that of hSI in terms of the expression of intestinal markers and genes involved in the defense response (Figure 4b). The expression profiles of WT Cont-hIOs, STAT3 KO Cont-hIOs, and STAT3 KO Mat-hIOs were tightly clustered together but not with that of hSI (Figure 4b).

Differentially expressed genes (DEGs) were divided into three clusters according to the differences in expression pattern, and the biological processes involving the genes of each cluster were analyzed through Gene Ontology (GO) analysis. Cluster 1 comprised highly expressed genes (1,439 genes) in WT Cont-hIOs, STAT3 KO Cont-hIOs, and STAT3 KO Mat-hIOs, in contrast to hSI and WT Mat-hIOs (Figure 4c). GO analysis of the genes in Cluster 1 revealed that many genes were involved in non-intestinal biological functions, such as chemical synaptic transmission, sodium ion transmembrane transport, and calcium ion transmembrane transport, and a few genes involved in cell adhesion and stem cell proliferation (Figure 4d). Cluster 2 comprised highly expressed genes (331 genes) in hSI and WT Mat-hIOs (Figure 4c). GO analysis demonstrated that the genes in Cluster 2 were not only involved in intestinal villus development, such as regulation of microvillus length, intermicrovillar adhesion, brush border assembly, regulation of microvillus assembly, and epithelial cell differentiation, but also played a role in intestinal processes, such as lipoprotein biosynthetic processes (*MTTP*, *APOB*, *APOA4*), intestinal absorption (*F11R*, *FABP1*, *TJP2*), intestinal D-glucose absorption (*PLS1*, *VIL1*), and mucus secretion (*VAMP8*, *AGR2*). The 420 DEGs belonging to Cluster 3 were highly expressed only in adult SI and did not increase or increased insufficiently after the in vitro maturation of WT hIOs (WT Mat-hIOs). Although some genes associated with epithelial cell development and intestinal cholesterol absorption were enriched in Cluster 3, they were mainly related to the function of the non-parenchymal cells of the human intestine, such as immune cells, muscle cells, and mesenchymal cells, involving processes such as immune response, T cell costimulation, immunoglobulin-mediated immune response, positive regulation of T cell activation, mesenchyme migration, and muscle contraction. These results suggest that the expression of Cluster 3 genes is generally low in in vitro matured hIOs due to low levels of non-parenchymal cells in hESC-derived hIOs at p2-5 used in this study (Supplementary Figure S3).

In addition, the STAT3-mediated signaling pathway was identified as a key signaling pathway regulating the Cluster 1 and Cluster 2 gene set by analyzing highly interconnected regions within functional interactome networks using the MCODE database (Figure 4e). Consistent with the fact that the expression patterns of Clusters 1 and 2 were not changed in STAT3 KO hIOs following in vitro maturation, the STAT3 signaling pathway was essentially required for the in vitro maturation of hIOs regardless of whether the direct IL-2 treatment or a co-culture system was used. Consistent with this result, gene set enrichment analysis (GSEA) of biological processes was performed to identify the functional biological pathways in WT Mat-hIOs compared with those of STAT3 KO Mat-hIOs. As a result, the pathways involved in intestinal absorption, epithelial cell differentiation, digestive system, and drug transport were the top-ranked subset signatures of WT Mat-hIOs (Supplementary Figure S4). Most importantly, the hierarchical clustering heatmap showed that WT Mat-hIOs and hSI expressed a variety of intestinal transporters and drug-metabolizing enzymes, which play critical roles in drug absorption and metabolism [28], whereas WT Cont-hIOs, STAT3 KO Cont-hIOs, and STAT3 KO Mat-hIOs did not fully recapitulate the expression levels of genes for intestinal transporters and drug-metabolizing enzymes (Figure 4f). This finding was further supported by data obtained in qPCR analyses of major intestinal transporters and drug-metabolizing enzymes, including GLUT2 (*SLC2A2*), GLUT5 (*SLC2A5*), PEPT1 (*SLC15A1*), P-gp (*ABCB1*), and OSTβ (*SLC51B*) (Figure 4g). The gene expression levels of these major glucose transporters, such as *GLUT2* (*SLC2A2*) and *GLUT5* (*SLC2A5*), were consistent with results obtained by the Fluo-4 AM assay to assess glucose transporter activity. Upon glucose stimulation, the intracellular Ca^2+^ release from the endoplasmic reticulum was impaired and exhibited significantly decreased Ca^2+^ transient amplitude (ΔF/F0) in WT Cont-hIOs, STAT3 KO Cont-hIOs, and STAT3 KO Mat-hIOs compared to WT Mat-hIOs (Figure 4h).

### 3.5. STAT3 KO hIOs Did Not Grow And Maintain upon In Vivo Transplantation

**Figure 5:**
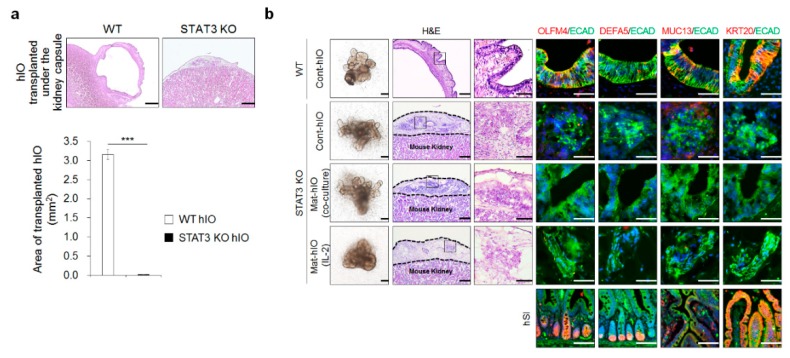
STAT3 KO led to the failure of in vivo growth and maintenance of hIOs. (**a**) H&E staining of hIOs after transplantation into the kidney capsule. Scale bar, 500 µm. Quantification of the area of transplanted hIOs (WT hIOs: n = 3, STAT3 KO hIOs; n = 6). Data are presented as the mean value of replicates ± SEM. *** *p* < 0.001 according to Student’s *t*-test. (**b**) H&E staining of transplanted STAT3 KO hIOs under the kidney capsule. The dotted line indicates where the transplanted hIOs is located. Scale bar, 200 µm. (High magnification, Scale bar, 50 µm). Immunofluorescence staining of the intestinal maturation markers in control, co-cultured, and IL-2-treated STAT3 KO hIOs and WT hIOs after maturation under the kidney capsule in vivo. Adult hSI tissues were used as the controls. Scale bar, 50 µm.

We aimed to examine whether STAT3 KO hIOs could acquire the characteristics of intestinal maturation upon in vivo transplantation, which is a widely used method to improve the maturity of tissues generated in vitro [29,30,31]. STAT3 KO hIOs were transplanted under the kidney capsule of immunodeficient NSG mice. The histologic examination showed that WT hIOs were successfully engrafted, however, STAT3 KO hIOs did not grow normally after in vivo transplantation under the kidney capsule of the recipient (Figure 5a). As a result, the transplanted STAT3 KO hIOs were much smaller than WT hIOs, and some degraded gradually following transplantation (Figure 5a). The markers of the mature intestinal epithelium, including OLFM4, DEFA5, MUC13, and KRT20, were detected only in the transplanted WT hIO epithelium after in vivo maturation and sections of adult hSI, whereas there were no OLFM4-, DEFA5-, MUC13-, or KRT20-positive cells in the transplanted STAT3 KO hIO epithelium positive for human-specific ECAD antibody regardless of whether in vitro maturation was induced (Figure 5b). Additionally, phospho-STAT3 (Y705) was observed in the WT hIO epithelium matured in vivo and sections of adult hSI (Supplementary Figure S5). Given that the STAT3 KO hIO epithelium failed to grow and maintain normally upon in vivo transplantation despite the normal formation of the epithelial and mesenchymal layers of STAT3 KO hIOs in vitro (Supplementary Figures S1 and S3) suggest that STAT3 activation is required for the successful engraftment and subsequent in vivo maturation of hIOs.

## 4. Discussion

The present results demonstrate that the selective pharmacological inhibition or genetic blockade of STAT3 by CRISPR-Cas9 technology effectively attenuates the in vitro maturation of epithelium of hIOs derived from hESCs induced by direct IL-2 treatment or a co-culture system using human T lymphocytes. Indeed, the expression levels of the intestinal maturation markers were significantly decreased or completely abolished after the treatment with STAT3 inhibitors or STAT3 KO. We also demonstrated that the activation of STAT3 as determined by STAT3 phosphorylation at Y705 is a hallmark of the in vitro-matured hIO epithelium. Genome-wide microarray analyses clearly showed that the majority of genes downregulated by STAT3 KO are associated with intestinal development and various intestinal functions. Therefore, our present results strongly support the hypothesis that the intracellular signaling of STAT3 is required for the induction of maturation signals via STAT3 phosphorylation and further downstream signaling within hIO epithelium.

Inducing in vitro maturation of hPSC-derived 3D organoids and differentiated cells from the fetal to adult stage remains a major challenge for many hPSC-derived models, which could be attributed to the absence of proper signals and/or the lack of a physiologically relevant microenvironment and other cellular systems surrounding the hSI that are important for intestinal maturation, such as immune function, functional vasculature, muscle cells, fibroblasts, and enteric nervous cells [32,33,34]. To imitate mature adult hSI, hPSC-derived hIOs require either in vivo transplantation of hIOs into immune-deficient mice [8,35] or in vitro maturation of hIOs by co-culturing them with human T lymphocytes or direct treatment of IL-2 [9]. Our study demonstrated that tyrosine phosphorylation of STAT3 is an intracellular event that may be important in the regulation of the in vitro maturation of hIO epithelium. The activation of intracellular STAT3 can induce hIO maturation independent of IL-2, as evidenced by the treatment of hIOs with IL-22, another activator of STAT3, or Colivelin, a chemical activator of STAT3 [9]. STAT3 functions in a cell type-specific manner by regulating distinct downstream targets [36]. STAT3 activity is necessary for the survival of small-intestine crypt stem cells [13]. STAT3 is also known to play a critical role in inflammatory bowel diseases (IBDs), with increased activation of STAT3 in some patients with active IBD as well as several autoimmune diseases [37,38]. In an animal model, the deletion of Stat3 in hematopoietic cells led to spontaneous colitis [39], and intestinal epithelial cell-specific Stat3-deficient mice exhibited severe chronic inflammation [40]. Epithelial STAT3 is also essential for mucosal wound healing responses [11], stem cell regeneration [12], host defense, and inflammation [41], implying that the delicate balance of STAT3-mediating signaling is crucial for intestinal homeostasis. Therefore, the pharmacological regulators of STAT3 could be considered as drug candidates for intestinal homeostasis and disease control since the inhibition of STAT3 may affect intestinal proliferation, maturation, and regeneration. Indeed, IL-22, also known as a potent activator of STAT3, is also known as a regulator of intestinal homeostasis and is being developed as a drug for intestinal diseases such as IBD [12,30].

In our microarray dataset, Cluster 2 included genes that were mainly involved in intestinal development and physiological functions, which were differentially downregulated in all STAT3 KO hIOs and WT Cont-hIOs compared with WT Mat-hIOs and hSI. Except for genes related to non-parenchymal cell function (Cluster 3), most DEGs (Clusters 1 and 2) in WT Mat-hIOs obtained by either co-culture with T lymphocytes or IL-2 treatment were changed in a manner similar to that of adult hSI. Many STAT3 downstream targets that are critical for cell survival, growth, cell cycle progression, and migration were downregulated in STAT3 KO hIOs [42,43,44]. A key signaling pathway that regulates the expression of these genes was identified as STAT 3-mediated signaling, and STAT3 KO hIOs did not respond to the maturation stimulus. However, further studies are needed to determine the precise mechanisms of STAT3 expression and phosphorylation in the context of hIO maturation.

Additionally, the absence of non-parenchymal cells in hIOs resulted in transcriptomic differences in the in vitro matured hIOs and adult hSI. Consistent with a previous report [4], early passage hIOs contained a mesenchymal layer, such as smooth muscle actin (SMA)^+^/desmin^+^ smooth muscle cells, vimentin^+^/SMA^+^ intestinal subepithelial myofibroblasts (ISEMFs), and desmin^+^/vimentin^+^ fibroblasts, in our system. However, as passaging of these hIOs proceeded, the mesenchymal layer of hIOs that contained non-parenchymal cells noticeably thinned and disappeared, and the intestinal non-parenchymal cells were not present in passage 2-4 hIOs used in this study, such as smooth muscle cells, ISEMFs, fibroblasts, and CD3^+^ and CD8^+^ immune cells. At this time point, hIOs mainly contained the intestinal epithelium. Therefore, for the acquisition of hIOs with gene expression profiles and physiological functions similar to adult hSI, applications of other specialized cell types, such as smooth muscle, the vascular system, the enteric nervous system, lymphatics and immune cells, and interstitial cells [45], will be needed.

Since hIOs can be made from the hPSCs of healthy controls and patients with an intestinal disease, mature hIOs may be a promising adult intestinal model for understanding human intestinal physiology and pathophysiology in a patient-specific manner. Therefore, the development of hIOs recapitulating the physiological functions of mature hSI is an important step toward providing a high-quality in vitro human intestine model. Here we report that the blockade of STAT3 by either its specific inhibitors or STAT3 KO hESCs fails to induce not only the in vitro maturation of hIOs, but also to maintain the in vivo transplanted hIOs. These results imply that STAT3 signaling contributes to the maturation of hPSC-derived hIOs. These findings highlight new mechanisms for controlling STAT3 in the in vitro maturation of hIOs and suggest new approaches for preparing human intestinal tissues from hPSCs. This ability may have important implications in several intestinal research fields, including development research, physiological and pharmacological studies, tissue engineering, and therapeutic transplantation.

In summary, our results demonstrated that co-cultured and IL-2-treated hIOs offer a promising approach for generating in vitro-matured human intestinal tissue mimics with appropriate physiological functionalities, although further work will be required to generate fully functional adult-like hIOs. Here, we present the potential usefulness of in vitro-matured hIOs in various aspects of in vitro applications and their potential use in vivo. This approach opens new avenues for facilitating the utilization of hIOs in drug evaluation, pharmacological testing, and the development of regenerative therapies for patients with intestinal failure.

## Supplementary Materials

The following are available online at https://www.mdpi.com/2077-0383/8/7/976/s1, Table S1: Oligonucleotides for in vitro transcription templates, Table S2: List of primers used in targeted deep sequencing, Table S3: List of the PCR primer sequences used in this study, Table S4: List of antibodies used in this study, Table S5: The expression level of genes known to be involved in the Intestinal markers, defense response, transporter ME, and STAT3 target genes, Figure S1: Immunofluorescence staining of WT hIOs and STAT3 hIO after in vitro maturation by either IL 2 treatment or co culture with stimulated Jurkat T lymphocytes, Figure S2: Immunofluorescence staining of WT hIOs , STAT3 inhibitor treated WT Mat hIOs , and STAT3 KO hIOs with an apoptosis specific marker (Cleaved caspase 3; Cl Casp3) and the intestinal epithelial marker, OLFM4 or LYZ, Figure S3: Immunofluorescence staining of hIOs for detecting mesenchymal population and immune cells, Figure S4: List of gene sets enriched in the in vitro matured WT hIOs (WT Mat hIOs) compared with the in vitro matured STAT3 KO hIOs (STAT3 KO Mat hIOs), Figure S5: Immunofluorescence staining for the phospho STAT3 (Y705) in WT Mat hIOs following in vivo maturation and hSI as controls. Scale bar, 50 µm.
