# Supplementary material for: Blockade of STAT3 Causes Severe In Vitro and In Vivo Maturation Defects in Intestinal Organoids Derived from Human Embryonic Stem Cells
Source: J Clin Med. 2019 Jul 4;8(7):976. doi: 10.3390/jcm8070976 (PMC6678857; doi:10.3390/jcm8070976)
Supplement: Supplementary file 1 [file jcm-08-00976-s001.pdf]

Supplementary Tables

Supplementary Table S1. Oligonucleotides for *in vitro* transcription templates.

Target	Sequence (5' to 3')
STAT3 sgRNA Forward	GAA ATT AAT ACG ACT CAC TAT AG TCTTGACTCTCAATCCAAG GTT TTA GAG CTA GAA ATA GCA AG
STAT3 sgRNA Reverse	AAAAAAGCACCGACTCGGTGCCACTTTTTCAAGTTGATAACGGACTAGCCTTATTTTAACTTGCTATTTCTAGCTCTAAAAC

Supplementary Table S2. List of primers used in targeted deep sequencing.

	Sequence (5' to 3')	
**Name**	**Forward**	**Reverse**
STAT3 1st PCR	TCCTCCCCAGAGCATCTTTA	GCCCAAGTAAATGAGGTCCA
STAT3 2nd PCR	TTTACAGTTGGGACCCCTGA	TCATTTTCCCCATCACCTGT
STAT3 deep	ACACTCTTTCCCTACACGACGCTCTTCCGATCTTTTACAGTTGGGACCCCTGA	GTGACTGGAGTTCAGACGTGTGCTCTTCCGATCTTCATTTTCCCCATCACCTGT

Supplementary Table S3. List of the PCR primer sequences used in this study.

Target gene	Primer (Forward)	Primer (Reverse)
GAPDH	GAAGGTGAAGGTCGGAGTC	GAAGATGGTGATGGGATTTC
CDX2	CTGGAGCTGGAGAAGGAGTTTC	ATTTTAACCTGCCTCTCAGAGAGC
SI	GGTAAGGAGAAACCGGGAAG	GCACGTCGACCTATGGAAAT
OLFM4	ACCTTTCCCGTGGACAGAGT	TGGACATATTCCCTCACTTTGGA
DEFA5	CCTTTGCAGGAAATGGACTC	GGACTCACGGGTAGCACAAC
KRT20	TGGCCTACACAAGCATCTGG	TAACTGGCTGCTGTAACGGG
MUC13	CGGATGACTGCCTCAATGGT	AAAGACGCTCCCTTCTGCTC
CREB3L3	ATCTCCTGTTTGACCGGCAG	GTCGTCAGAGTCGGGGTTTG
DPP4	TGGAAGGTTCTTCTGGGACTG	CAGCTGTAGCATCATCTGTGCC

LCT	CTGCAGGCCTCAACAAGTCT	GCCAAAAGGCGTCATCTTCA
OCT4	GAGGAGTCCCAGGACATCAA	AATAGAACCCCCAGGGTGAG
NANOG	CAAAGGCAAACAACCCACTT	ATTGTTCCAGGTCTGGTTGC
SLC2A2	GGCCAGCAGGTTCATCATCAGCAT	CCTTGGGCTGAGGAAGAGACTGTG
SLC2A5	CGCCAAGAAAGCCCTACAGA	GCGCTCAGGTAGATCTGGTC
SLC15A1	TGCTCAGGGGATAACCAAAG	ACCTGCCAGGAGCACGTC
ABCB1	GCCAAAGCCAAAATATCAGC	TTCCAATGTGTTCGGCATTA
SLC51B	GAGCTGCTGGAAGAGATGCT	TGCTTATAATGACCACCACAGC
CHGA	TGACCTCAACGATGCATTTC	CTGTCCTGGCTCTTCTGCTC
GIP	GTACGCGGAAGGGACTTTCA	AGTCCCGCAGCAAATCTTCA

Supplementary Table S4. List of antibodies used in this study.

Antibodies	Catalog No.	Company	Dilution
**Pluripotency markers**			
anti-OCT4	sc-9081	Santa Cruz	1:100 for IF
anti-NANOG	AF1997	R&D systems	1:40 for IF
anti-SSEA-3	MAB1434	Millipore	1:30 for IF
anti-SSEA-4	MAB1435	Millipore	1:30 for IF
anti-TRA-1-60	MAB4360	Millipore	1:100 for IF
anti-TRA-1-81	MAB4381	Millipore	1:100 for IF
**Intestinal organoid differentiation markers**			
anti-CDX2	ab15258	abcam	1:100 for IF
anti-KLF5	ab137676	abcam	1:100 for IF
anti-SOX9	sc-7314	Santa Cruz	1:50 for IF
anti-Villin1	sc-7672	Santa Cruz	1:50 for IF
anti-Mucin2	sc-7314	Santa Cruz	1:50 for IF
anti-Chromogranin A	MA5-14536	Thermo Scientific	1:200 for IF
anti-Lysozyme	ab76784	abcam	1:200 for IF

anti-E-Cadherin	610182	BD Biosciences	1:200 for IF
anti-E-Cadherin	AF648	R&D systems	1:500 for IF
**Intestine maturation markers**			
anti-alpha 5 Defensin	ab90802	abcam	1:50 for IF
anti-OLFM4	ab85046	abcam	1:100 for IF
anti-DPPIV			
anti-LCT			
**STAT3 signaling markers**			
anti-STAT3	4904	CST	1:1000 for WB
anti-phospho STAT3 (Y705)	ab76315	abcam	1:2000 for WB
**Mesenchyme markers**			
anti-α-SMA	A5228	Sigma	1:500 for IF
anti-Desmin	AB907	Chemicon	1:50 for IF
anti-Vimentin	Sc7557	Santa Cruz	1:100 for IF
**Immune cell markers**			
anti-CD3-FITC	300306	BioLegend	1:100 for IF
anti-CD8-FITC	344704	BioLegend	1:100 for IF
**Western blot**			
anti-β-actin	A5441	Cell Signaling	1:2000 for WB

Supplementary Table S5. The expression level of genes known to be involved in the Intestinal markers, defense response, transporter ME, and STAT3 target genes

Intestinal marker

Gene Symbol	WT Mat-hIO (co-culture)	WT Mat-hIO (IL-2)	STAT3 KO Cont-hIO	STAT3 KO Mat-hIO (co-culture)	STAT3 KO Mat-hIO (IL-2)	hSI	Systematic
ALPI	1.135	1.567	0.422	0.591	0.741	134.233	A_23_P337658
BMI1	0.276	0.370	1.304	1.409	1.351	0.840	A_23_P314115
CA1	8.063	25.664	1.479	1.361	1.420	35.607	A_23_P168916
CDX2	2.901	2.702	0.080	0.060	0.172	3.718	A_33_P3304501
CFTR	0.303	0.135	0.135	0.085	0.187	4.707	A_23_P215720
CHGA	7.400	10.593	1.185	1.031	1.153	7.388	A_33_P3293164
CROT	1.349	1.521	1.428	1.151	1.379	2.284	A_23_P168669
FABP2	21.028	41.234	1.479	1.361	10.140	301.717	A_23_P391711
G6PC	1.804	1.844	1.479	2.348	3.405	1812.148	A_23_P385017
GUCY2C	58.681	65.499	0.346	0.392	2.944	36.313	A_23_P76312
HNF4A	11.769	18.943	1.041	0.958	1.108	11.715	A_23_P28761
IFT140	0.546	0.727	1.890	1.532	1.629	0.337	A_23_P140725
ISX	4.773	6.849	0.018	0.027	0.377	4.633	A_32_P217140
KRT20	26.206	49.069	1.715	0.889	5.230	42.430	A_23_P66854
KRT8	8.682	8.943	1.038	0.601	1.116	13.641	A_23_P14072
LGR5	2.931	1.049	1.367	1.471	0.391	1.194	A_23_P98974
MGAM	1.338	2.976	0.863	0.513	0.993	91.092	A_23_P42897
MUC13	45.080	54.133	0.335	0.213	3.947	19.412	A_23_P155236
MUC2	1.174	2.381	1.382	1.818	1.076	100.852	A_33_P3412384
OLFM4	1.676	4.806	1.726	0.227	1.372	980.527	A_24_P181254
PYY	1.198	1.293	1.479	1.361	1.420	3.455	A_33_P3329958
SI	0.560	3.269	0.050	0.046	2.996	57.223	A_32_P302205
SOX9	1.542	1.458	0.944	1.041	0.889	0.814	A_23_P26847
TFF3	6.166	6.794	0.655	0.612	0.610	4.806	A_23_P393099
VIL1	8.063	10.602	0.151	0.294	0.650	6.974	A_23_P16866

Defense

Gene Symbol	WT Mat-hIO (co-culture)	WT Mat-hIO (IL-2)	STAT3 KO Cont-hIO	STAT3 KO Mat-hIO (co-culture)	STAT3 KO Mat-hIO (IL-2)	hSI	Systematic
ALLC	0.613	0.249	0.599	0.155	0.162	0.335	A_23_P108734
ANG	4.227	6.219	0.156	0.154	0.378	0.838	A_33_P3236177
C3orf33	0.599	0.536	1.128	0.926	0.996	0.857	A_23_P301476
CD48	0.428	0.218	0.327	0.229	0.239	26.914	A_32_P175934
CD74	1.295	1.220	0.157	0.145	0.151	83.131	A_23_P70095
CD83	0.056	0.052	0.270	0.357	0.647	0.769	A_23_P70670
CD84	1.198	1.293	1.479	1.361	1.420	14.071	A_23_P361940
CLEC5A	1.198	1.293	1.479	1.361	1.420	38.067	A_23_P304356
CST3	3.237	3.576	0.483	0.596	0.873	3.738	A_33_P3228266
CX3CL1	0.265	0.340	0.955	0.702	0.822	1.393	A_24_P390495
CYSLTR1	1.137	1.639	1.728	0.656	3.018	11.533	A_23_P22660
DEFA5	808.269	1568.930	1.479	2.236	1.420	149691.420	A_23_P112086
DEFA6	247.719	350.957	2.214	1.361	1.420	153309.810	A_24_P363711
DEFB1	9.449	9.655	6.646	3.154	5.297	64.761	A_23_P71480
ERG	1.198	3.142	1.479	1.361	4.486	9.711	A_23_P301414
GATA3	0.281	0.308	13.521	5.812	4.869	0.917	A_33_P3360341
HSF1	0.994	1.119	1.267	0.914	1.044	1.093	A_23_P253841
ICOSLG	4.062	4.316	1.479	1.361	1.420	8.391	A_23_P317667
IL17RB	2.049	1.559	0.377	0.576	0.648	3.057	A_24_P157370
IL32	8.219	12.372	0.195	0.179	1.691	28.598	A_23_P15146
INHBA	0.611	0.388	0.402	0.098	0.348	0.814	A_23_P122924
INHBB	0.016	0.010	1.284	0.881	0.947	0.356	A_23_P153964
KCNN4	21.185	15.510	1.056	0.712	0.947	5.918	A_23_P67529
KREMEN1	1.030	0.852	1.977	1.348	1.733	2.703	A_23_P68851
LILRA2	1.198	1.293	1.479	1.361	1.420	10.072	A_23_P142205
LILRB3	0.496	0.820	3.727	2.403	2.702	69.907	A_32_P70158
LILRB5	1.198	1.293	1.479	1.361	1.420	1628.486	A_23_P4773
LSP1	4.076	4.957	1.612	1.492	1.822	6.017	A_33_P3369567
MLF2	1.087	1.222	0.933	0.787	0.965	1.045	A_23_P13873
MPO	1.178	1.271	1.454	1.338	1.396	2.881	A_23_P141173
MST1R	14.608	16.725	1.071	1.123	1.876	20.688	A_23_P256312
MX1	1.214	1.706	0.875	0.505	1.958	28.656	A_23_P17663
MX2	0.838	0.880	1.720	1.487	1.607	8.807	A_33_P3278200
NLRP1	6.632	1.293	3.138	2.695	1.551	65.382	A_23_P89550
NLRP3	0.200	0.216	1.678	1.423	1.834	6.752	A_33_P3281695

NOD1	1.408	1.357	1.006	0.653	0.794	2.773	A_24_P129277
PTPRCAP	0.940	1.603	1.809	1.738	1.346	33.327	A_33_P3295056
REG3A	141.504	452.817	5.511	2.110	1.420	135279.310	A_23_P119936
RELA	1.317	1.412	0.486	0.340	0.486	0.903	A_33_P3209433
RNASE6	17.881	19.349	0.219	0.146	0.274	31.195	A_23_P3014
SOCS6	1.357	1.549	0.853	1.000	1.030	0.813	A_23_P207981
SP140	2.027	2.542	2.797	2.736	3.012	30.529	A_24_P328504
TAP1	1.693	1.411	0.884	0.808	0.925	10.152	A_23_P59005
TAPBP	3.410	3.313	1.065	0.743	1.052	8.081	A_23_P259580
TFF3	18.310	22.918	0.589	0.529	0.556	4.298	A_33_P3334305
TIAL1	1.203	1.652	1.233	0.853	0.914	0.658	A_23_P161488
TLR1	1.283	1.155	1.066	0.287	0.543	24.810	A_23_P10873
TLR3	2.598	3.077	0.748	0.461	0.646	10.557	A_23_P29922
TMEM8B	0.447	0.550	1.379	1.827	1.760	0.833	A_24_P317827
TNF	0.504	1.584	0.697	0.572	2.145	15.094	A_23_P376488
TNFRSF1A	3.496	4.161	0.875	0.707	1.156	4.087	A_23_P139722
TNIP1	2.641	3.348	0.876	1.079	1.333	2.599	A_23_P30435
TPSAB1	1.198	1.293	1.479	1.361	1.420	643.277	A_23_P37702
WAS	1.341	1.305	2.186	2.113	1.827	24.561	A_23_P96331

Transporter ME

Gene Symbol	WT Mat-hIO (co-culture)	WT Mat-hIO (IL-2)	STAT3 KO Cont-hIO	STAT3 KO Mat-hIO (co-culture)	STAT3 KO Mat-hIO (IL-2)	hSI	Systematic
ABCB1	8.045	11.262	1.500	0.315	0.382	21.127	A_23_P82523
ABCB11	1.198	4.493	3.256	1.361	1.728	3.393	A_23_P39703
ABCB4	0.780	0.753	1.135	0.902	1.235	0.615	A_33_P3336760
ABCC2	7.307	13.256	1.408	3.759	15.803	37.244	A_23_P44569
ABCC3	14.291	16.096	0.230	0.808	0.632	10.451	A_33_P3298024
ABCG2	2.724	5.888	1.604	1.972	2.428	94.111	A_33_P3361067
ATP1A1	3.755	3.951	0.613	0.663	0.804	7.662	A_23_P1072
CYP2C19	38.186	67.237	0.653	5.253	6.684	37.928	A_33_P3326075
CYP2C9	30.430	51.270	0.678	4.232	5.239	28.025	A_23_P12767
CYP2J2	9.984	11.970	0.850	0.552	1.366	67.620	A_23_P103486
CYP3A4	0.741	1.495	0.832	0.726	0.849	878.209	A_33_P3251342
CYP3A5	45.125	60.083	1.526	2.807	6.534	60.397	A_23_P8801
GSTK1	3.204	3.962	0.397	0.264	0.446	7.533	A_23_P20107
SLC10A1	1.198	1.293	1.479	1.361	1.420	18.065	A_23_P25698
SLC15A1	21.518	37.858	1.618	1.592	9.163	90.844	A_33_P3373273
SLC16A1	2.263	2.757	1.545	1.257	1.170	0.754	A_33_P3282434
SLC22A11	1.198	1.551	1.479	1.361	1.420	11.585	A_23_P47199
SLC22A12	0.463	0.723	0.182	0.208	0.175	0.361	A_33_P3316721
SLC22A2	1.198	8.482	1.479	1.361	1.420	2.930	A_23_P111395
SLC22A3	3.593	4.505	1.516	0.763	1.617	1.414	A_32_P309404
SLC22A5	2.682	2.801	0.881	0.851	1.092	6.704	A_24_P174755
SLC22A7	1.431	1.599	2.036	1.978	1.844	4.654	A_23_P93217
SLC22A8	1.323	1.448	1.274	1.015	1.139	15.107	A_33_P3367171
SLC2A5	0.835	11.197	0.754	0.371	3.933	1841.547	A_23_P160159
SLC36A1	5.107	6.078	0.611	0.735	0.775	1.916	A_33_P3251093
SLC51A	16.376	24.028	0.254	0.105	1.254	2.514	A_24_P385732
SLC51B	22.021	35.390	1.153	0.565	2.707	80.907	A_23_P436284
SLC6A20	2.022	2.194	1.698	1.361	2.094	6.103	A_24_P20369
SLC9A3	0.819	0.759	1.133	0.799	1.606	9.801	A_23_P30582
SLCO1B1	2.252	2.232	1.237	0.695	1.010	0.170	A_23_P128254
SLCO2B1	17.838	21.429	0.214	0.683	1.306	26.035	A_23_P150768
SULT1B1	44.078	59.124	0.592	0.555	4.148	26.037	A_23_P302470
SULT1E1	2.583	3.682	0.274	0.297	2.156	4.275	A_23_P155786
SULT2A1	19.447	28.807	2.477	1.506	6.291	1074.706	A_23_P67381
UGT1A6	5.235	9.095	1.479	2.565	2.122	456.160	A_24_P222872
UGT1A8	3.999	12.101	1.479	2.061	3.249	454.174	A_33_P3302075

Color index

The ratios are color-coded, as indicated by the color index bar. Red: Upregulated genes compared to undifferentiated control; blue: downregulated genes compared to undifferentiated control.

Fold	Color
>10	
>7.5	
>5	
>3	
>2	
>1.75	
>1.5	
>1.25	
1	
<0.8	
<0.6666666	
<0.5714286	
<0.5	
<0.3333333	
<0.2	
<0.1333333	
<0.1	

Supplementary Figures

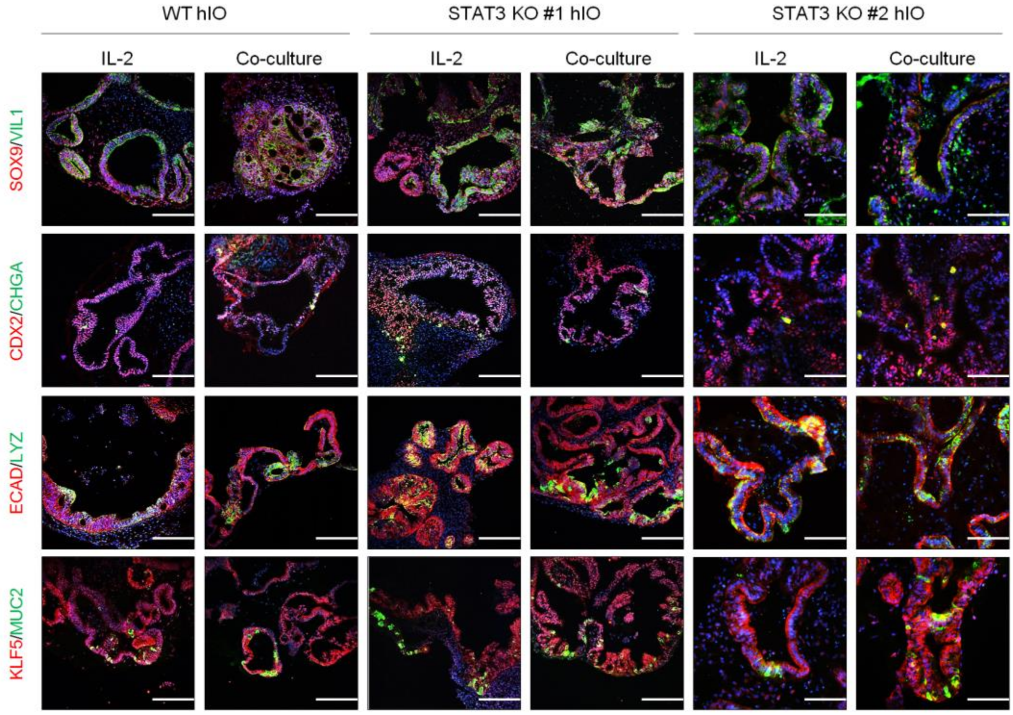

Supplementary Figure S1. Immunofluorescence staining of WT hIOs and STAT3 hIO after *in vitro* maturation by either IL-2 treatment or co-culture with stimulated Jurkat T lymphocytes for intestine-specific markers (SOX9, CDX2, and KLF5), enterocyte marker (VIL), enteroendocrine cell marker (CHGA), Paneth cell marker (LYZ), goblet cell marker (MUC2), and epithelial marker (ECAD). Nuclei were stained with DAPI (blue). Scale bar, 200 μm.

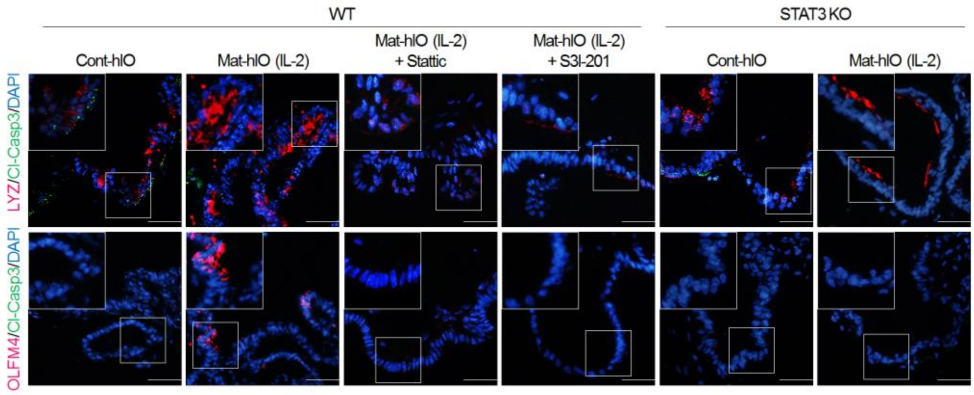

Supplementary Figure S2. Immunofluorescence staining of WT hIOs, STAT3 inhibitor treated WT Mat-hIOs, and STAT3 KO hIOs with an apoptosis-specific marker (Cleaved caspase 3; Cl-Casp3) and the intestinal epithelial marker, OLFM4 or LYZ. Scale bar, 50 μm

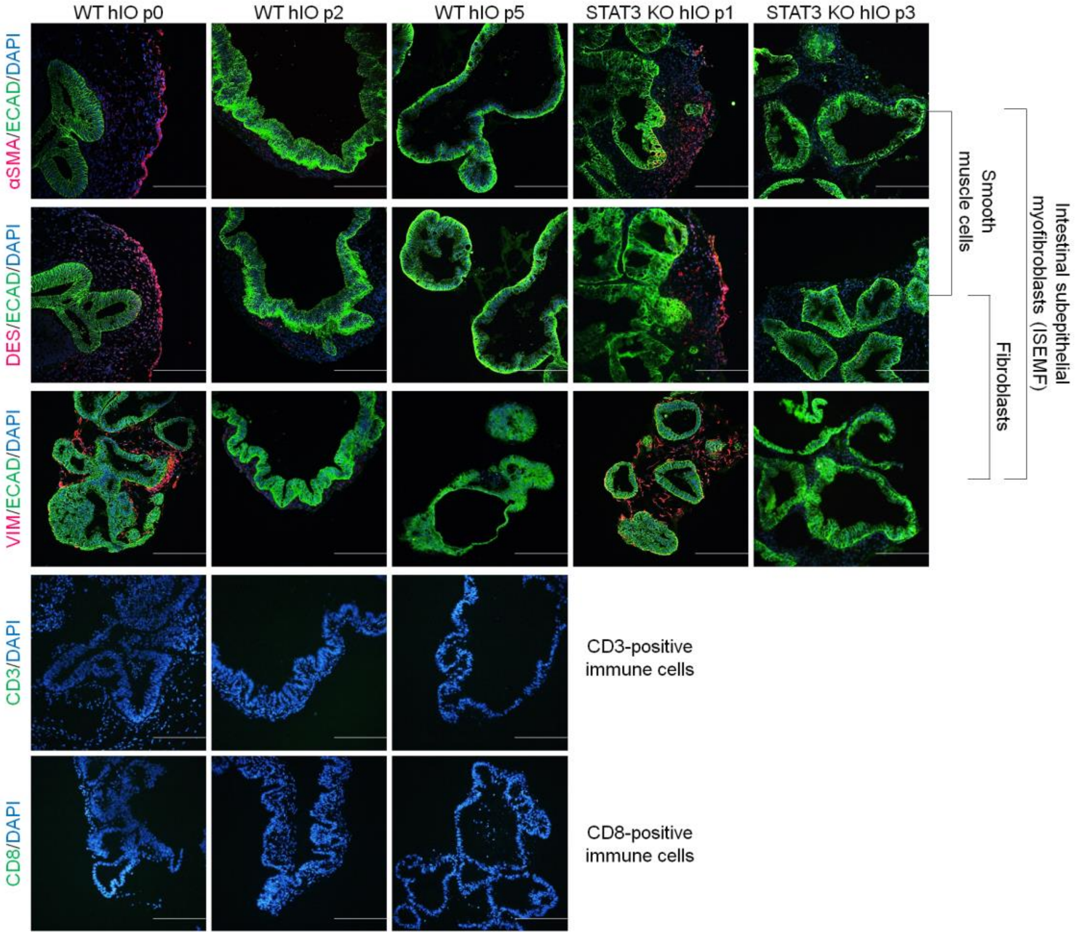

Supplementary Figure S3. Immunofluorescence staining of hIOs for detecting mesenchymal population and immune cells. Non-parenchymal cells were not present in WT hIOs at p2-5, STAT3 hIO at p3 such as smooth muscle cells, ISEMFs, fibroblasts and $CD3^+$ and $CD8^+$ immune cells. Nuclei were stained with DAPI (blue). Scale bar, 100 μm.

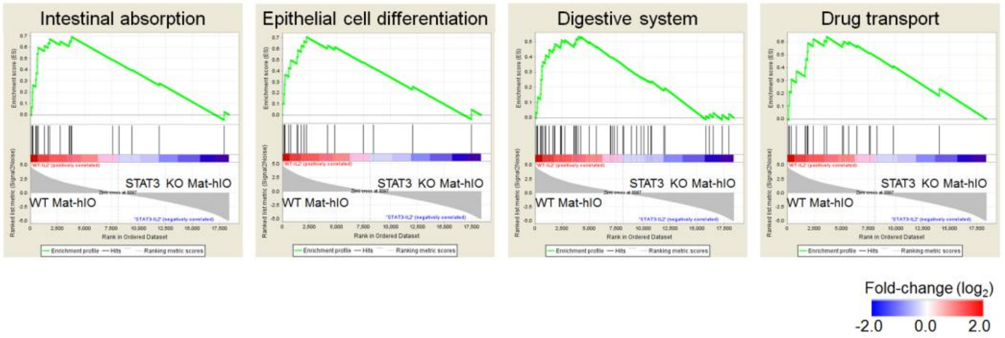

Supplementary Figure S4. List of gene sets enriched in the in vitro matured WT hIOs (WT Mat-hIOs) compared with the in vitro matured STAT3 KO hIOs (STAT3 KO Mat-hIOs). Enrichment plot of the top ranked subset signature by BP (biological process); intestinal absorption, epithelial cell differentiation, digestive system and drug transport.

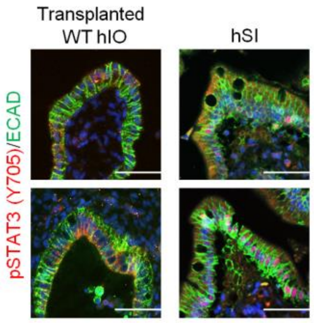

Supplementary Figure S5. Immunofluorescence staining for the phospho-STAT3 (Y705) in WT Mat-hIOs following *in vivo* maturation and hSI as controls. Scale bar, 50 µm.
